# White paper on J Young Pharm Statistics JYP: Where Do We Stand !!!

**DOI:** 10.4103/0975-1483.62204

**Published:** 2010

**Authors:** Ahmed KK Mueen

**Affiliations:** *Editor, J Young Pharm, E-mail: jyp@inpharm.org*

Six months, four issues, processed 231 manuscripts, indexed and abstracted in more than 18 databases and many more.

*Journal of Young Pharmacists* (JYP) (an addendum to InPharm Communiqué) is an official quarterly Journal published by the InPharm Association, URL: www.inpharm.org (a Young Pharmacists Group of India). JYP is a peer-reviewed open-access quarterly journal featuring research articles of exceptional significance in all areas of pharmaceutical sciences. JYP provides comprehensive coverage of issues affecting pharmaceutical education and career.

Having recently completed the first volume with the launch of *Journal of Young Pharmacists* (Oct–Dec, 2009; Issue 4), we have seen JYP become ever more internationally represented in its published articles, and at the same time have seen its tremendous growth, attributable mainly to global submissions. Growth by itself in a short period of time, assuming that quality is maintained, is not necessarily bad. In fact, we are proud that JYP is one of the preferred choices for publication in the field of pharmacy. JYP gives the reader an excellent opportunity to access the cutting-edge pharmaceutical literature at no cost.

JYP is currently indexed and abstracted in Chemical Abstracts, Caspur, DOAJ, EBSCO Publishing’s Electronic Databases, Expanded Academic ASAP, Genamics JournalSeek, Global Health, Google Scholar, Health and Wellness Research Center, Health Reference Center Academic, Hinari, Index Copernicus, OpenJGate, SCOLOAR, SIIC databases and Ulrich’s International Periodical Directory.

**Table:** Journal of Young Pharmacists – Performance Chart Release dates: First Issue: 6^th^ June, 2009; Second Issue: 10^th^ August, 2009; Third Issue: 13^th^ October, 2009; Fourth Issue: 10^th^ January, 2010

Submitted to first decision for the year 2009	
Number of articles submitted	231
Days to suggest reviewers	5.94 (0, 50)
Days taken by reviewers	16.60 (0, 82)
Days taken by Editor for decision	4.80 (0, 49)
Days until paper is under review	27.34

**Table:** 

Revisions for the year 2009	
Days from first decision until revision arrives	10.16 (0, 61)
Days for Editor to take decision	2.45 (0, 7)
Articles rereviewed	16
Days for rereview by reviewers	9.75 (0, 20)
Days to send revision decision by Editors	2.45 (0, 7)
Days from revision receipt to revision decision	14.65

**Table:** 

Accepted papers for the year 2009	
Days from first submission to acceptance	53.35 (0, 115)
Days from acceptance to publication	86.30 (14, 149)

Total articles submitted for the year 2009

**Table:** Total 231 articles submitted in 2009

Article type	Submitted	Accepted (%)	Rejected (%)	Under review (%)
Editorial	1	1 (100)	0 (0)	0 (0)
General articles	13	5 (38)	8 (62)	0 (0)
Invited article	1	1 (100)	0 (0)	0 (0)
Letter to Editor	2	1 (50)	1 (50)	0 (0)
Obituary	0	0	0	0
Original article	103	39 (38)	54 (52)	10 (10)
Pharm analysis	14	6 (43)	5 (36)	3 (21)
Pharm chemistry	7	2 (29)	2 (29)	3 (43)
Pharm management	0	0	0	0
Pharm marketing	2	2 (100)	0 (0)	0 (0)
Pharmaceutics	20	11 (55)	2 (10)	7 (35)
Pharmacognosy	8	6 (75)	1 (13)	1 (13)
Pharmacology	13	11 (85)	1 (8)	1 (8)
Total decisions	159	85 (53)	74 (47)	
Total articles	184	85 (46)	74 (40)	25 (14)

**Table:** Country-wise manuscript submissions

Country	Manuscripts submitted
Bangladesh	1
Brazil	2
India	217
Iran	2
Libya	1
Malaysia	2
Nigeria	2
Pakistan	3
Vietnam	1

**Table:** Results for the year 2009

Total number of authors registered with the site	363
Number of authors who have submitted manuscripts	187
Number of authors who have submitted more than one manuscript	9
Number of manuscripts from abroad	14 (6%)
Number of original articles from abroad	5 (5%)

**Table:** Country-wise referees

Country	Manuscripts reviewed
Bangladesh	1
Chin	1
India	90
Ireland	1
Jordan	1
Libya	1
Saudi Arabia	2
Singapore	1
Spain	1
USA	2

Publishing is always a cost. A sense of the pressing concern of maintaining revenues to cover the expenses of JYP is one of the tasks ahead. We humbly request all the stakeholders and principals of pharmacy colleges to get subscribed JYP in their library in order to strengthen the journal.

I add a note of thanks to Dr. DK Sahu (Medknow Publications and Media Pvt. Ltd.) and his excellent team members, without whose cooperation and support and untiring response to my follow-ups, it would not have been possible to launch new issues on time.

